# Comparable *in vitro* Function of Human Liver-Derived and Adipose Tissue-Derived Mesenchymal Stromal Cells: Implications for Cell-Based Therapy

**DOI:** 10.3389/fcell.2021.641792

**Published:** 2021-03-26

**Authors:** Furkan Yigitbilek, Sabena M. Conley, Hui Tang, Ishran M. Saadiq, Kyra L. Jordan, Lilach O. Lerman, Timucin Taner

**Affiliations:** ^1^William J. von Liebig Center for Transplantation and Clinical Regeneration, Mayo Clinic, Rochester, MN, United States; ^2^Division of Nephrology and Hypertension, Mayo Clinic, Rochester, MN, United States; ^3^Department of Immunology, Mayo Clinic, Rochester, MN, United States

**Keywords:** Mesenchymal stroma/stem cells, liver, adipose tissue, cellular senescence, angiogenesis

## Abstract

Mesenchymal stem/stromal cells (MSCs) have been investigated extensively for their immunotherapeutic and regenerative properties, which may differ by cell source. In MSCs harvested from donors matched for sex, age, and body mass index, we compared the proliferative and migration functions of liver-derived MSCs (L-MSCs) and adipose tissue-derived MSCs (A-MSCs) (*n* = 6 donors each). Cellular senescence was evaluated by senescence-associated beta-galactosidase enzyme activity and expression of senescence-associated secretory phenotype (SASP) factors using real-time quantitative polymerase chain and by western blot assay. The pro-angiogenic and reparative potency of MSCs was compared by co-culturing MSCs with injured human umbilical vein endothelial cells (HUVEC). The proliferation and migration properties were similar in L-MSCs and A-MSCs. Although cell cycle arrest and SASP genes were similarly expressed in both MSCs, tumor necrosis factor alpha gene and protein expression were significantly downregulated in L-MSCs. In co-cultured injured HUVEC, A-MSCs restored significantly more tubes and tube connections than L-MSCs. Therefore, despite many functional similarities between L-MSCs and A-MSCs, L-MSCs have enhanced immunomodulatory properties, while A-MSCs appear to have better pro-angiogenic and vascular reparative potency. Availability of a broad range of cellular options might enable selecting cell-based therapy appropriate for the specific underlying disease.

## Introduction

Mesenchymal stem/stromal cells (MSCs) are non-hematopoietic multipotent cells with the inherent ability to self-renew and differentiate into tissues of mesenchymal origin (Dominici et al., 2006). They have essential roles in tissue repair and regeneration through their anti-apoptotic activity and stimulation of angiogenesis, as well as notable immunomodulatory activities (Han et al., 2019). MSCs have been demonstrated to regulate immune reactivity through paracrine mechanisms by inhibiting both innate and adaptive responses (Zhou et al., 2019). Their impact on lymphocytes, macrophages, dendritic cells, and natural killer (NK) cells has been investigated extensively (Wang et al., 2014; Najar et al., 2016). Owing to these potent immunomodulatory properties, MSCs are actively investigated as cellular therapeutics in multiple disease processes.

Mesenchymal stem/stromal cells have been successfully isolated from several tissues, including fat, placenta, peripheral blood, and liver since their first discovery, and while MSCs from different sources have many functional similarities, they also show several differences (Mattar and Bieback, 2015; Kozlowska et al., 2019). Given the liver’s unique tolerogenic microenvironment (Taner et al., 2016, 2017, 2018), we had postulated that liver MSCs (L-MSCs) are superior immunomodulators than their counterparts isolated from other tissues. In fact, we demonstrated that L-MSCs inhibited alloreactive T cell proliferation better than MSCs isolated from adipose tissue (A-MSC) and bone marrow (BM-MSC) (Taner et al., 2020). Transcriptome analysis also demonstrated that the L-MSCs have significantly upregulated expression of genes and gene sets associated with immune regulation. Therefore, L-MSCs may be better candidates for cellular therapies.

However, besides immunomodulation, other properties might determine the functional potency of MSC. Among the most beneficial functions of MSCs are their angiogenic and tissue repair properties. MSC-based cellular therapy has been beneficial in various disease conditions, including coronary artery disease and skin wound repair through regulation of angiogenesis and tissue repair (Fan et al., 2020). These MSC functions are strongly influenced by the premature aging of the cells, termed cellular senescence (Suvakov et al., 2019). Cellular senescence is defined as a state of cell cycle arrest mediated by the tumor suppressor proteins p53, p21, and p16 that alters the cellular functions. Cells respond to the senescence state by activating the senescence-associated secretory phenotype (SASP) (Krtolica and Campisi, 2002). The SASP is characterized by diverse growth factors, cytokines, chemokines, and matrix metalloproteinases and might alter the cell metabolism, influence tissue homeostasis, and compromise the immunoregulation of MSCs (Lunyak et al., 2017). Senescence characteristics of MSCs have been shown to differ based on their tissue origin (Turinetto et al., 2016). Therefore, we believe the proper use of MSCs for clinical applications requires a general understanding of the functions and senescence characteristics of MSCs from different sources.

While tissue source accounts for key differences in the immunomodulatory properties of MSCs generated *in vitro*, whether other functions of L-MSC are similarly altered remain unknown (Yoo et al., 2009; Waldner et al., 2018). This is particularly important as variations in tissue microenvironment within the same tissue also influence MSCs. For example, we have recently shown that A-MSCs obtained from obese individuals undergo early senescence compared to A-MSCs from age-matched non-obese individuals, which was linked to decreased function (Conley et al., 2020). However, whether intrinsic cellular senescence in L-MSC differs from A-MSCs remains unknown, especially given the well-known superior regenerative capacity of the liver compared to other organs. Thus, we aimed to compare different properties of L-MSCs, which might affect their potential therapeutic applications.

To that end, we have tested the proliferative capacity, migratory activity toward inflammatory signals, response to stress signals, and reparative function on blood vessels of L-MSCs in comparison to A-MSCs.

## Materials and Methods

### Isolation and Culture of MSC

The study was approved by the Mayo Clinic Institutional Review Board. MSCs were isolated from liver tissue of deceased organ donors (L-MSC) and subcutaneous abdominal adipose tissue of living kidney donors and weight reduction surgery (A-MSC). These donors underwent screening and met the criteria for organ donation. All samples were processed within 8 h of procurement. Liver and adipose tissues were minced in a Petri dish and mixed with 0.075% Collagenase IV (STEMCELL Technologies, Cambridge, MA, United States) in Dulbecco’s phosphate-buffered saline. After 45 min, enzyme action was stopped by adding platelet lysate (PL5%) MSC media; Advanced Minimum Essential Medium (Thermo Fisher Scientific, Waltham, MA, United States), PLTGold Human Platelet Lysate (EMD Millipore, Burlington, MA, United States), and GlutaMAX (Thermo Fisher Scientific, Waltham, MA, United States). Digested tissue was centrifuged, resuspended in fresh media, and filtered twice before plating in PL5% MSC media. Non-adherent cells were removed every 3 days thereafter. Cultures were maintained at 37°C, 5% CO_2_ in a humidified incubator.

### MSC Characterization

Cell morphology, trilineage differentiation, and phenotype were analyzed to characterize MSCs according to The International Society for Cellular Therapy (Dominici et al., 2006). Briefly, MSCs’ differentiation ability into adipocyte, osteocyte, and chondrocyte lineages was assessed using the Human MSC Functional Identification Kit (R&D Systems, Minneapolis, MN, United States), according to the manufacturer’s recommended protocol. Differentiation was tested by immunofluorescent staining using anti-mFABP4 (adipocyte), anti-human osteocalcin (osteocyte), and anti-human Aggrecan (chondrocyte).

The detailed characterization of both the L-MSC and the A-MSC used in the current study was reported previously (Conley et al., 2020; Taner et al., 2020). Both types of MSCs demonstrated plastic-adherent characteristics and spindle-shaped morphology when cultured in PL5% MSC media. Furthermore, the cells were labeled with fluorochrome-conjugated monoclonal antibodies to confirm canonical MSC markers (CD73, CD90, and CD105) expression, as well as non-expression of CD14 and CD45 by flow cytometry. Additionally, CD200 and CD274 expressions were tested for L-MSC. L-MSC and A-MSC data were analyzed using Kaluza software (Beckman Coulter, Chaska, MN, United States) and Analysis Software (IDEAS version 6.2), respectively.

### MSC Migration and Proliferation

Cellular proliferation of MSCs was evaluated by a live cell analysis and imaging system (Incucyte^®^, Sartorius, Ann Arbor, MI, United States). Approximately 2.5×10^3^ A-MSCs or L-MSCs (*n* = 6 each type) were seeded per well in a 96-well plate, then placed into Incucyte^®^ SX1 Live-Cell Analysis System at 37°C in a CO_2_ incubator and allowed to propagate in culture for 48 h. During incubation, MSCs’ images were taken every 2 h, and proliferation was analyzed using Incucyte^®^ Cell-by-Cell Analysis Software. Migratory functions of MSCs were tested using a QCM^TM^ Colorimetric Cell Assay (EMD Millipore, Burlington, MA, United States) as well as a scratch assay, according to the manufacturer’s instructions. Briefly, for colorimetric cell assay, 1.5×10^5^ MSCs (*n* = 6 each type) were seeded into the insert of 24 wells transwell system, and PL5% MSC media was added to the lower chamber, then MSCs were incubated 24 h at 37°C in a CO_2_ incubator. Afterward, the insert was stained, and the stain then extracted. The optical density of the extracted stain was measured at 560 nm.

The migration scratch assay was performed in two independent experiments. A 5×10^5^ L-MSCs (*n* = 5) and A-MSCs (*n* = 5) were seeded on a 24-well plate as duplicates. The semi-automated BioTek AutoScratch^TM^ Wound Making Tool has created scratches in confluent cell monolayers, and then wells were washed twice to remove detached cells before placing them on BioTek Cytation^TM^ 5 Cell Imaging Multi-Mode Reader, which was maintained at 37°C and 5% CO_2_ for 24 h. Images were taken at 2-h intervals to observe their movements which were analyzed by BioTek Scratch Assay App. The change in the scratch area was calculated every 2 h and normalized to the baseline scratch area. The migration speed was calculated individually for cells until the scratch closed, and the average migration speed was calculated by taking the mean of their individual speeds.

Chemoattractants used in the migration assay were those enriched in human platelet lysate, including stromal cell-derived factor-1 (SDF-1), interleukin-8 (IL-8), monocyte chemoattractant protein-1 (MCP-1), and platelet-derived growth factor-1 (PDGF-1).

### Senescence and Senescence-Associated Secretory Phenotype (SASP) Marker Expression by Quantitative Polymerase Chain Reaction (qPCR)

Using the mirVana^TM^ PARIS kit (Thermo Fisher Scientific, Waltham, MA, United States), total RNA was isolated from MSCs according to the kit protocol. RNA concentration was measured using a NanoDrop spectrophotometer and the first-strand cDNA produced by the Superscript^TM^ VILO^TM^ cDNA synthesis kit (Thermo Fisher Scientific, Waltham, MA, United States). Relative qPCR was performed on a QuantStudio 7 Real-Time PCR system using TaqMan^®^ assays. The fold change of gene expressions was calculated using the 2-ΔΔCT method. All TaqMan^®^ probes were purchased from Thermo Fisher Scientific. Cellular senescence was determined by the expression of the cell cycle arrest markers p16 (Cat.# HS00923894), p21 (Cat.# HS00355782), and p53 (Cat.# HS01034249), as well as the SASP markers, activin A (INHBA, Cat.# HS01081598), monocyte chemoattractant protein-1 (MCP-1, HS00234140), plasminogen activator inhibitor-1 (PAI-1, Cat.# HS00167155), interleukin-1 alpha (IL-1α, Cat.# HS00174092), interleukin 6 (IL-6, Cat.# HS00174131), and tumor necrosis factor alpha (TNFα, Cat.# HS00174128). Gene expression was normalized to TATA-binding protein (TBP, Cat.# HS00427620).

### Western Blot Assay

Mesenchymal stem/stromal cells were detached with Tryple^TM^ (Gibco^TM^, Invitrogen, Carlsbad, CA, United States) and centrifuged to obtain a pellet. Then pellets were lysed for 20 min on ice with cell lysis buffer (Cell Signaling Technology, Inc., Danvers, MA, United States), and total proteins transferred on 4–20% SDS-PAGE gels and transferred onto PVDF membranes as duplicates. The membranes were blocked with 5% BSA for an hour and incubated with primary antibodies. After washes, the membranes were incubated with secondary antibodies for an hour at room temperature. The membranes were washed and then incubated with ECL Western Blot Substrate (Cell Signaling Technology, Inc., Danvers, MA, United States) and were visualized on ImageQuant^TM^ LAS 4000. Anti-TNFα (Cat# ab6671) and PAI-1 (Cat# ab66705) antibodies were purchased from Abcam (Cambridge, MA, United States). GAPDH antibody was used to normalize the results. All experiments were done separately and independently for each cell line.

### Senescence-Associated Beta-Galactosidase Enzyme Activity

Senescence-associated beta-galactosidase (SA-β-Gal) enzyme activity, a participant in cellular senescence (Dimri et al., 1995), was evaluated using the assay of the β-galactosidase enzyme (Enzo, Farmingdale, NY, United States) and staining (Dojindo Molecular Technologies, Inc., Rockville, MD, United States) according to manufacturer’s instructions. The percentages of β-gal positive cells were quantified in 3 fields of view using the Cytation-5 Cell Imaging Reader. Galactosidase beta 1 (GLB1, Cat.# HS01035168), the encoding gene for SA-β-Gal, expression was evaluated using qPCR.

### Co-culture of MSCs With Human Umbilical Vein Endothelial Cells

Co-culture experiments were performed to evaluate the pro-angiogenic and reparative potency of the MSCs, as previously described (Conley et al., 2020). In brief, human umbilical vein endothelial cells (HUVEC, Cell Applications, San Diego, CA, United States) were grown in endothelial cell growth medium (EGM^TM^-Plus Endothelial Cell Growth Media-Plus BulletKit^TM^ Medium, Lonza, Cohasset, MN, United States), and seeded at a density of 3.5×10^5^ cells/well in the lower chamber of a transwell plate. The cells were divided into 4 groups to test the effect of MSCs. Group 1 was the control group and was cultured in normal conditions, whereas groups 2–4 were co-incubated with TNFα (10 ng/mL) and transforming growth factor-beta 1 (TGF-β1, 5 ng/mL) for 3 days to induce cellular injury (Khan et al., 2017). After the co-incubation, this media was changed with fresh growth medium, subsequently co-cultured with either L-MSCs (group 3) or A-MSCs (group 4) (1.75×10^5^ cells/well insert) for another 24 h. HUVEC were then harvested, lysed, and prepared for qPCR analyses.

Following treatment and co-culture procedures (as described above), HUVEC were plated onto a Matrigel^®^ matrix-coated plate (CORNING, Corning, NY, United States) at a final concentration of 7×10^4^ cells/500 μL and incubated overnight in a cell culture incubator. Zeiss Axio Observer microscope was used to determine the number of tube-like structures and connections in five different fields of view for each well.

### Statistical Analysis

Normally distributed data were represented as mean ± standard deviation and non-normal data as median and interquartile range. Comparisons among groups were performed using the two-sample *t*-test with a 5% type-I error. One-way analysis of variance (ANOVA) was employed to detect differences in co-culture experiments, whereas cellular proliferation data were analyzed using repeated ANOVA. All data were considered significant if *p* ≤ 0.05. Statistical analysis was accomplished using JMP software (SAS Institute, Cary, NC, United States).

## Results

### MSC Culturing and Characterization

All the cells used in the current study had been isolated and characterized in our previous studies independently. The six primary L-MSC batches tested herein were generated from deceased donor liver allografts (Taner et al., 2020). Table 1 summarizes the demographics of each group. A-MSC from six subjects matched for sex, age, and body mass index (BMI) were harvested from fat tissue (Conley et al., 2020). The mean age of the L-MSC and A-MSC donors was similar, as was their mean BMI. After harvesting and isolating, MSCs were expanded in culture for three passages to prepare for the experiments. Culture media was replaced every 3 days until MSCs reached 80% confluence throughout this study.

**TABLE 1:** L-MSC from liver tissue of deceased organ donors and A-MSC from subcutaneous abdominal adipose tissue of living kidney donors and weight reduction surgery were matched for sex, age, and body mass index (*n* = 6 per group).

	**L-MSC (*n* = 6)**	**A-MSC (*n* = 6)**	***P*-value**
Sex (M/F (ratio))	3/3 (50%)	3/3 (50%)	
Age (years ± SD)	44 ± 18	48 ± 15	0.37
Body mass index (kg/m^2^± SD)	31.2 ± 5.9	35.1 ± 5.8	0.14

*The results are presented as the mean ± standard deviation, and statistical significance was analyzed by the two-sample *t*-test. MSC, Mesenchymal stem/stromal cell; L-MSC, Liver MSC; A-MSC, Adipose MSC.*

### Migratory and Proliferative Capacities of MSC

**FIGURE 1:**
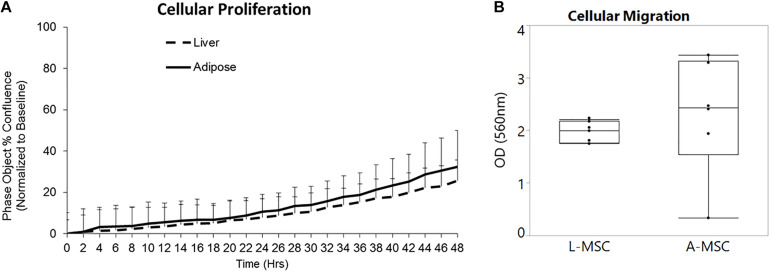
L-MSCs and A-MSCs demonstrate similar proliferative and migration functions *in vitro*. **(A)** To compare the proliferative capability of MSCs, cells were incubated for 48 h and evaluated by a live-cell analysis and imaging system. There was no significant difference at none of the time points between MSCs (*p* = 0.10). **(B)** The migratory function toward chemoattractants was tested using colorimetric cell assay. The migratory function of L-MSCs and A-MSCs was similar (*p* = 0.24). The results are presented as the mean ± standard deviation. Statistical significance was analyzed by repeated ANOVA for cellular proliferation and the two-sample *t*-test for cellular migration. MSC, Mesenchymal stem/stromal cell; L-MSC, Liver MSC; A-MSC, Adipose MSC.

**FIGURE 2:**
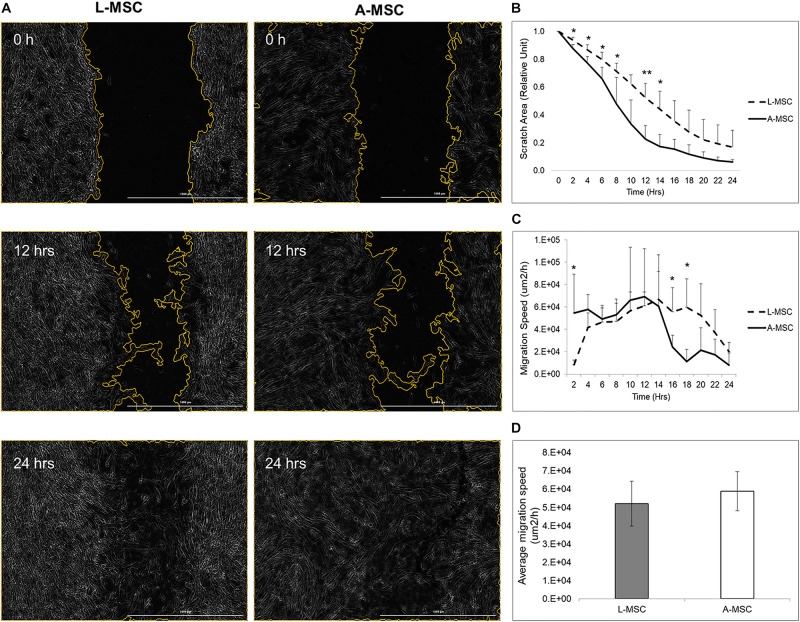
MSC migration was assayed by BioTek AutoScratch^TM^ Wound Making Tool, with cells incubated and observed under BioTek Cytation^TM^ 5 Cell Imaging Multi-Mode Reader. **(A)** Representative images were obtained immediately, 12 h, and 24 h after the scratch. **(B)** Quantified scratch area closure by A-MSCs and L-MSCs. The relative unit was calculated from the time of scratch creation. **(C)** The cell migration speed, calculated using scratch areas, plotted at 2-h intervals. **(D)** The average migration speed was calculated to the point when the MSCs’ reached their confluences. The data presented as mean ± standard deviation and statistical significance was analyzed by the sample *t*-test (*p < 0.05, **p < 0.005). L-MSCs: Liver MSCs, A-MSCs: Adipose MSCs.

In order to compare the proliferation capability of L-MSCs and A-MSCs, cells were incubated for 48 h and assessed with live-cell imaging. At none of the time points was there a difference in percent confluence, demonstrating similar proliferate kinetics in both cell types (*p* = 0.10) (Figure 1A). Two methods were employed to assess cellular migration. When the migration of the MSCs toward chemoattractants across a semi-permeable membrane was tested, A-MSCs were noted to have variable migratory capacity, consistent with our previous data (Conley et al., 2020). Migration kinetics of L-MSCs were more homogenous. On average, the migratory function of L-MSCs and A-MSCs was similar (*p* = 0.24) (Figure 1B). Moreover, we performed a migration scratch assay for 24 h to further characterize their migratory kinetics (Figure 2A). The closure of scratch was significantly faster for A-MSCs than L-MSCs for the first 14 h of the assay (Figure 2B). A-MSCs had reached their peak migration speed after 10–12 h, while L-MSCs reached theirs after 14–16 h. Initially, A-MSCs’ migration speed was significantly higher than L-MSCs, which decreased substantially as the cells became confluent. Thus, L-MSCs’ migration speed was significantly higher than A-MSCs at the 16th and 18th hours of the assay (Figure 2C). However, their average migration speed until they reached confluence was similar (*p* = 0.45) (Figure 2D).

### Expressions of Senescence and SASP Markers

**FIGURE 3:**
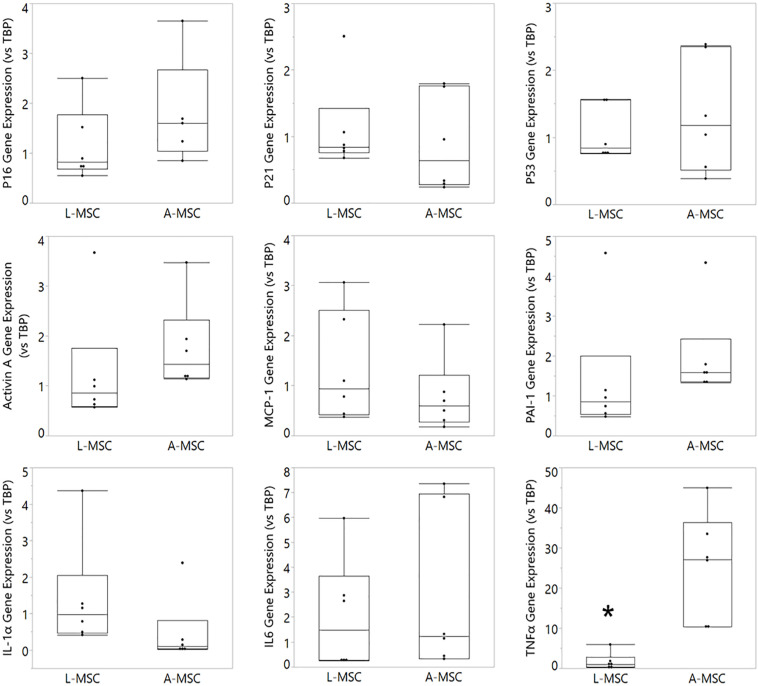
Comparative analysis of senescence and SASP marker expression on L-MSC and A-MSC. Cellular senescence was determined by the expression of p16, p21, and p53. SASP markers were determined by the expression of activin A, MCP-1, PAI-1, IL-1α, IL-6, and TNFα. Gene expressions were normalized to TBP and calculated using the 2-ΔΔCT method. Data are presented as the mean ± standard deviation, and statistical significance was analyzed by the sample *t*-test (*p < 0.005 vs. A-MSC). MSC, Mesenchymal stem/stromal cell; L-MSC, Liver MSC; A-MSC, Adipose MSC; MCP-1, Monocyte chemoattractant protein-1; PAI-1, Plasminogen activator inhibitor-1; IL-1α, Interleukin-1 alpha; IL-6, Interleukin 6; TNFα, Tumor necrosis factor alpha; TBP, TATA-binding protein; SASP, Senescence-associated secretory phenotype; qPCR, Quantitative Polymerase Chain Reaction.

Cellular senescence is a stress response mechanism that leads to irreversible cell cycle arrest, and senescence in MSCs impairs their function (Turinetto et al., 2016). Senescence in L-MSCs vs. A-MSCs was compared by testing the relative expression of cell cycle arrest (p16, p21, and p53) and canonical SASP markers (activin A, MCP-1, PAI-1, IL1A, IL6, TNFα) using qPCR. There was no difference between the L-MSCs and A-MSCs in the expression of p16, p21, and p53. Yet, the L-MSCs had significantly lower expression of the pro-inflammatory TNFα gene (*p* = 0.003) compared to the A-MSCs (Figure 3).

**FIGURE 4:**
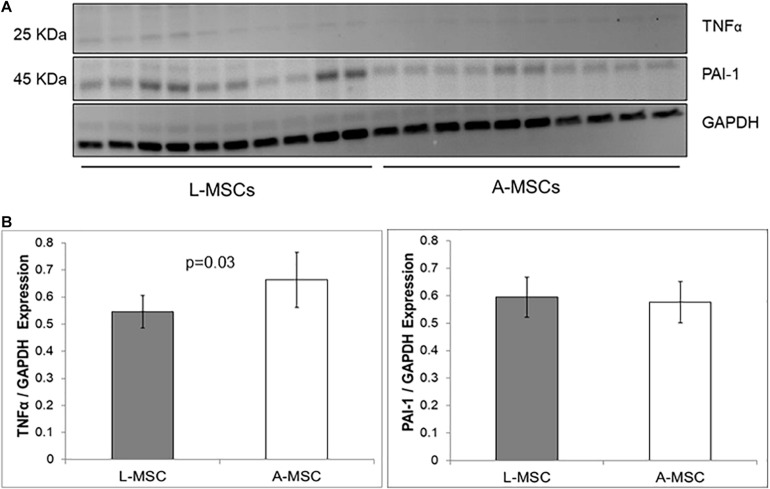
Comparison of SASP markers protein expression by western blot assay. **(A)** The western blot gel images of TNFα and PAI-1 are shown. The gels were run under the same experimental conditions, one blot for each cell line. **(B)** TNFα and PAI-1 expressions in both MSC types. Protein expressions were quantified by GAPDH antibody. TNFα expression is significantly lower levels in L-MSCs (*p* = 0.03). Data are presented as the mean ± standard deviation and statistical significance analyzed by the sample *t*-test. L-MSCs, Liver MSCs; A-MSCs, Adipose MSCs; TNFα, Tumor necrosis factor alpha; PAI-1, Plasminogen activator inhibitor-1; GAPDH, Glyceraldehyde 3-phosphate dehydrogenase.

The protein expression of two representative SASP markers, TNFα and PAI-1, was tested with Western blot (Figure 4A). Similar to the qPCR results, PAI-1 protein expression was comparable in L-MSC and A-MSC (*p* = 0.7), whereas TNFα expression was significantly lower in L-MSCs than A-MSCs (*p* = 0.03) (Figure 4B).

### Lysosomal Activity of L-MSC

**FIGURE 5:**
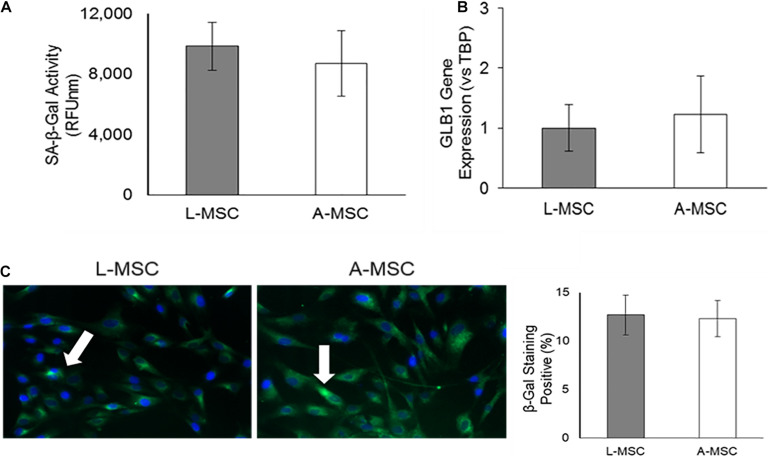
Lysosomal activity in MSCs. SA-β-Gal is a lysosomal enzyme that participates in cellular senescence, and its activity was investigated both at the enzyme and gene levels. **(A)** L-MSCs and A-MSCs have similar enzyme activity (*p* = 0.16). **(B)** qPCR was performed on the GLB1. GLB1 gene expression was similar between L-MSCs and A-MSCs. **(C)** The percentage of β-galactosidase stained cell nuclei was not different between MSCs [white arrows indicate apoptotic nuclei (green), counterstained with DAPI (blue)]. The results are presented as the mean ± standard deviation, and statistical significance was analyzed by the two-sample *t*-test. MSC, Mesenchymal stem/stromal cell; L-MSC, Liver MSC; A-MSC, Adipose MSC; SA-β-Gal, Senescence-associated beta-galactosidase; GLB1, Galactosidase beta 1.

Because the cellular response to stress correlates with DNA damage and subsequent increase in lysosomal activity, next, we investigated the SA-β-Gal enzyme activity both at the enzyme (Figure 5A) and gene (Figure 5B) levels. SA-β-Gal enzyme activity and gene expression of GLB1, which encodes the SA-β-Gal enzyme, was similar in L-MSCs and A-MSCs. Likewise, both MSC subjects displayed similarly β-galactosidase stained apoptotic signals (Figure 5C).

### Characterization of L-MSC Protective and Pro-Angiogenic Properties

**FIGURE 6:**
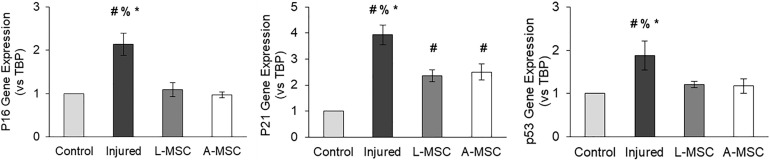
HUVEC were incubated with TNFα and TGF-β1 for 3 days to induce injury. qPCR was performed on injured HUVEC co-cultured with MSCs to analyze cell cycle arrest. Upregulation of p16, p21, and p53 gene expression on injured HUVEC confirmed the injury. Gene expressions were normalized to TBP and calculated using the 2-ΔΔCT method. The results are presented as the mean ± standard deviation, and statistical significance was analyzed by the two-sample *t*-test (*p* < 0.05 # vs. Control, % vs. L-MSC, * vs. A-MSC). MSC, Mesenchymal stem/stromal cell; L-MSC, Liver MSC; A-MSC, Adipose MSC; TBP, TATA-binding protein.

Next, we tested the protective and angiogenic properties of L-MSCs in comparison to A-MSCs. HUVEC were injured by incubating them with TNFα and TGF-β1 for 3 days, and the injury was confirmed by the upregulation of cell cycle markers. The expression of the cell cycle makers in injured HUVEC was reduced down to the baseline level when they were co-cultured with either L-MSCs or A-MSCs for 24 h (*p* < 0.0001) (Figure 6).

**FIGURE 7:**
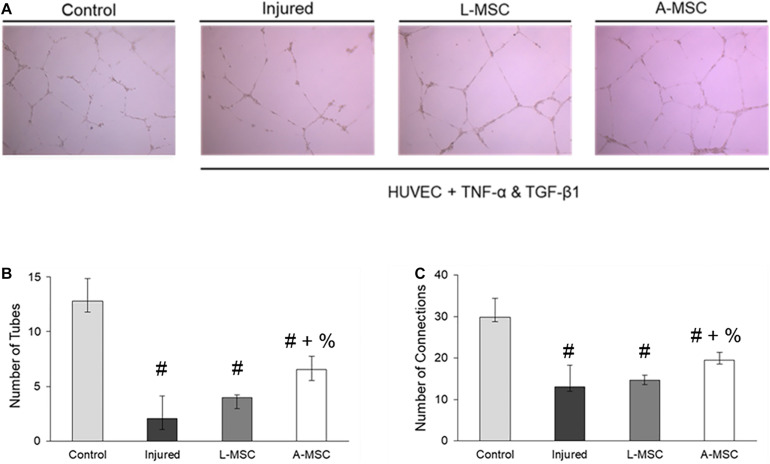
The pro-angiogenic and reparative potency of L-MSCs and A-MSCs on HUVEC. **(A)** The representative images show tube formation under different conditions. The numbers of tubes **(B)** and tube connections **(C)** were decreased in injured HUVEC and increased after co-culture with MSCs, but more increased with A-MSCs more than L-MSCs. The results are presented as the mean ± standard deviation, and statistical significance was analyzed by the sample *t*-test (*p* < 0.05 # vs. Control, + vs. Injured, % vs. L-MSC). MSC, Mesenchymal stem/stromal cell; L-MSC, Liver MSC; A-MSC, Adipose MSC; HUVEC, Human umbilical vein endothelial cells; TNFα, Tumor necrosis factor alpha; TGF-β1, Transforming growth factor-beta 1.

The pro-angiogenic capacity of MSCs was tested by tube formation capacity of injured HUVEC. The analysis demonstrated that after HUVEC were injured, their tube formation was significantly blunted; however, the co-culture of injured HUVEC with A-MSCs increased the number of tubes and tube connections more than L-MSCs (Figure 7), suggesting greater pro-angiogenic potency.

## Discussion

In attempt to identify the liver components that contribute to its tolerogenic microenvironment, we have recently isolated and expanded human L-MSCs *in vitro*. To characterize the cellular properties of L-MSCs and identify their unique properties relative to MSCs derived from other tissues, here we compared them to the commonly investigated and well-characterized A-MSCs. Interestingly, we found that the proliferative kinetics, migratory functions, and intrinsic senescence characteristics of L-MSCs are comparable to those of A-MSCs.

The liver is a highly metabolic organ with unique immunoregulatory functions through its location at a crossroads of the portal and systemic blood circulation. This strategic position allows it to carry out its tolerogenic immune function, clearing gut-derived nutrients, antigens from old cells, and bacterial degradation (Zheng and Tian, 2019). A combination of uniquely tolerogenic liver-resident immune cells and the liver’s sinusoidal microanatomy contribute to the tolerogenic microenvironment of the liver (Thomson and Knolle, 2010; Dou et al., 2018; Abrol et al., 2019). Guided by the liver’s unique tolerogenic characteristics, we had previously postulated that L-MSCs would possess immunomodulatory properties superior to their counterparts isolated from other tissues (Taner et al., 2020). In fact, our earlier studies demonstrated that L-MSCs inhibit alloreactive T-cell proliferation and also suppress the frequency of IFNγ-producing alloreactive T-cells more effectively than A-MSCs. At steady state, the transcriptome of L-MSCs expanded *in vitro* is remarkably different from that of A-MSCs, in that immunomodulatory genes and genesets are enriched in L-MSCs compared to A-MSCs (Taner et al., 2020). Consistent with our previous studies, both the gene and protein expression of TNFα were significantly lower in L-MSCs. As a known proinflammatory cytokine, TNFα plays a crucial role in many chronic inflammatory diseases (Davignon et al., 2018). Therefore, our study supports the notion that L-MSCs, with their immunomodulatory properties, might be better candidates for cellular therapeutics in inflammatory conditions. MSCs from different tissue sources have been reported to have variable phenotypes, transcriptomes, secretomes, and functions (Strioga et al., 2012). For example, A-MSCs possess better proangiogenic properties than BM-MSCs (Kozlowska et al., 2019). Given that L-MSCs are conceptually more difficult to harvest than A-MSCs, it is important to compare them rigorously to A-MSCs to justify their use as cellular therapeutics and establish their proliferation, migration, and vasculoprotective properties.

Clinical applications of MSCs depend on the successful homing of the cells to the target sites. When tissue damage occurs, resident MSCs are activated, and some MSCs are released into circulation and home to the injury site (Chapel et al., 2003; Caplan, 2009). Systemic administration of MSCs, however, has not resulted in reliable engraftment of MSCs in the injury sites in multiple pre-clinical and clinical trials (Barbash et al., 2003; Devine et al., 2003). Such delivery of MSCs results in entrapment of most of the cells within the pulmonary capillaries (Barbash et al., 2003; Devine et al., 2003; Scarfe et al., 2018). However, targeted administration of MSCs into the site of inflammation can, conceptually, overcome this problem. MSCs delivered locally might then migrate through the tissue via the chemoattractant gradient (Ullah et al., 2019). Mimicking this gradient *in vitro*, here we found that the L-MSCs possess similar migratory properties and kinetics as A-MSCs.

The similarity between the two types of MSCs was also notable in their proliferation kinetics. *In vitro* expansion of MSCs may gradually lead to cellular senescence and arrest of the cell cycle, mimicking responses to stress (Baxter et al., 2004). Importantly, cellular senescence involving cell-cycle arrest impairs MSC characteristics, particularly the regenerative and immunomodulatory properties of MSCs, which in turn transforms MSCs from an immunomodulatory to SASP-releasing pro-inflammatory phenotype (Lunyak et al., 2017). Gnani et al. (2019) showed that senescent BM-MSCs have a reduced inhibitory effect on the proliferation of peripheral mononuclear cells than younger BM-MSCs. Also, senescent MSCs negatively affect their niche by activating pro-inflammatory gene expression, which leads to decreased hematopoietic stem cells’ clonogenic potential (Gnani et al., 2019). As such, senescence characteristics of MSC have important clinical and safety implications. We, therefore, compared the molecular changes underlying senescence and cell proliferation between A-MSCs and L-MSCs generated under the same culturing conditions and harvested at the same passage, cell cycle arrest markers were quantified. Our results indicate similar expression of genes that contribute to cell cycle arrest (p16, p21, and p53), as well as the SASP markers in both types of MSCs. Furthermore, the protein expression of the SASP marker PAI-1 was similar. The upregulation of the SA-β-Gal enzyme, encoded by the GLB1 gene, is also a marker for cellular senescence (Lee et al., 2006). Our cohort also showed no difference in lysosomal activity at both the enzymatic and gene levels between the two MSC types.

We and others have previously shown that co-incubation of HUVEC with TNFα and TGF-β induces cellular injury, evidenced by cell cycle arrest and senescence (Khan et al., 2017). Indeed, we found that the expression of cell cycle arrest markers was upregulated in injured HUVEC. Co-culture with MSC from both tissue sources reversed the upregulation of cell cycle arrest markers injured HUVEC Additional testing of their pro-angiogenic activity using *in vitro* formation of tube-like networks (DeCicco-Skinner et al., 2014), however, demonstrated that while both MSCs types were able to partly protect the function of injured HUVEC, A-MSCs facilitated the generation of more tubes and tube-like networks.

The present study has several limitations due to the overall small sample size. However, in order to minimize the age-, sex-, and obesity-related differences in MSC, the cell donors were well-matched. Both donor groups were also in the obese range, which might have affected MSC functions (Conley et al., 2020). Furthermore, albeit not statistically significant, A-MSCs were obtained from individuals with slightly higher BMI, which could have impacted our results. Our results would need to be validated in further, larger studies.

In conclusion, we demonstrate that the L-MSCs have comparable proliferative and migratory profiles to those of A-MSCs. Similarly, senescence markers of L-MSCs do not differ from A-MSCs cultured *in vitro* under the same conditions. While L-MSCs have been shown to possess superior immunomodulatory properties, A-MSCs appear to be better in angiogenesis and repair of injured endothelial cells. Therefore, MSC-based therapy needs to be tailored to the underlying disease state. These findings provide further evidence for the safety of L-MSCs in their potential utilization as cellular therapeutics in disease processes with underlying inflammation. Further studies are needed to compare their reparative and angiogenic effects *in vivo*.

## Data Availability Statement

The original contributions presented in the study are included in the article/supplementary material, further inquiries can be directed to the corresponding author.

## Author Contributions

FY, SC, LL, and TT contributed to the conception and design of the study. FY, SC, HT, IS, KJ, LL, and TT collected the data, contributed to the data analysis, and interpretation. TT and LL provided financial support. FY wrote the first draft of the manuscript. All authors contributed to manuscript revision, read and approved the submitted version.

## Conflict of Interest

LL is an advisor to AstraZeneca and Janssen Pharmaceuticals. The remaining authors declare that the research was conducted in the absence of any commercial or financial relationships that could be construed as a potential conflict of interest.
